# Osteopontin Expression in Thyroid Cancer: Deciphering EMT-Related Molecular Mechanisms

**DOI:** 10.3390/biomedicines9101372

**Published:** 2021-10-01

**Authors:** Bruna Prunes Pena Baroni Viana, Amanda Vitória Pampolha Gomes, Etel Rodrigues Pereira Gimba, Luciana Bueno Ferreira

**Affiliations:** 1 Grupo de Hemato-Oncologia Molecular, Coordenação de Pesquisa, Instituto Nacional de Câncer, Praça da Cruz Vermelha, 23, 6° andar, Rio de Janeiro 20230-130, CEP, Brazil; brunabaroniviana@hotmail.com (B.P.P.B.V.); avitoriapam@gmail.com (A.V.P.G.); 2 Programa de Pós-Graduação Stricto Sensu em Oncologia, Instituto Nacional de Câncer, Rua André Cavalcanti, 37, 3° andar, Rio de Janeiro 20231-050, CEP, Brazil; 3 Centro de Ciências Biológicas e da Saúde, Instituto Biomédico, Universidade Federal do Estado do Rio de Janeiro, Rua Frei Caneca, 94, Rio de Janeiro 20211-010, CEP, Brazil; 4 Departamento de Ciências da Natureza, Universidade Federal Fluminense, Rua Recife 1-7, Bela Vista, Rio das Ostras 28880-000, CEP, Brazil; 5 Programa de Pós-Graduação em Ciências Biomédicas, Fisiologia e Farmacologia, Instituto Biomédico, Av. Prof. Hernani Melo, 101, Niterói 24210-130, CEP, Brazil

**Keywords:** thyroid cancer, osteopontin, epithelial–mesenchymal transition

## Abstract

Thyroid cancer is the most common tumor arising from the endocrine system and generally presents good prognosis. However, its aggressive subtypes are related to therapeutic resistance and early metastasis. Epithelial–mesenchymal transition (EMT) and its reverse process, the mesenchymal–epithelial transition (MET), are key events mediating cancer progression, including in thyroid cancer. The matricellular protein osteopontin (OPN) has been reported as a master regulator of EMT in many tumor types. Although high OPN expression has been described and associated with important aspects of thyroid cancer progression, there is no clear evidence regarding OPN as a regulator of EMT in thyroid cancer. Thus, taking together the known roles of OPN in the modulation of EMT in cancer and the information reporting the expression of OPN in thyroid tumor progression, this review aims at summarizing and discussing data related to EMT in thyroid cancer and its putative relation to the roles of OPN in the development of thyroid cancer. These data provide new insights into the molecular mechanisms by which OPN could potentially modulate EMT in thyroid tumors, generating evidence for future studies that may contribute to new therapeutic, prognostic and/or diagnostic tools.

## 1. Introduction

Thyroid tumors are the most common malignancies of the endocrine system, representing the fifth most prevalent cancer worldwide [1]. The thyroid gland can originate different histopathological tumor subtypes. Thyroid follicular cells can give rise to the well-differentiated papillary thyroid carcinoma and follicular thyroid carcinoma, the most common types, with the best prognostic value. In addition, these cells can originate anaplastic thyroid carcinoma, classified as an undifferentiated type, as well as poorly differentiated thyroid carcinoma, both of which are responsible for poor prognosis and therapy resistance. Otherwise, thyroid parafollicular or C-cells cause medullary thyroid carcinoma, which is characterized by early metastasis [2].

Epithelial–mesenchymal transition (EMT) is a pivotal process that is activated in many physiological systems. In embryogenesis, cells tend to be remodeled, altering their morphology between epithelial and mesenchymal-like features, in order to migrate, colonize and develop the different embryonic leaflets, in addition to organ formation [3]. This process is well described during the development of cancer, in which tumor cells often lose their epithelial phenotype while mesenchymal markers are upregulated, favoring higher migration, invasion and metastasis rates. The reverse process, called mesenchymal–epithelial transition (MET), is also pivotal to the establishment of metastatic sites. Growing evidence has shown that these two events are highly plastic and dynamic, generating intermediate or partial EMT phenotypes, which are important for tumor progression [3,4].

Among several regulators of EMT in tumors, osteopontin (OPN) has been described as a key modulator of this process. OPN is a glycophosphoprotein that is involved in physiological and pathological conditions and can regulate EMT in many cancer types, such as breast, ovarian, lung, liver and gastric carcinomas [5]. OPN and its splice variants (OPN-SV), mainly osteopontin-a (OPNa), osteopontin-b (OPNb) and osteopontin-c (OPNc), are known to activate diverse signaling pathways that are related to tumor progression features [6,7]. Recently, the expression of the osteopontin-4 (OPN4) and osteopontin-5 (OPN5) splice variants was also described [8], although their functional roles in cancer progression remain unclear.

Thyroid cancer displays high OPN expression levels, which are related to tumor development features [9]. Further, thyroid tumor development and progression are related to EMT-associated signaling pathways [10]. However, despite the actual data regarding OPN and its splice variants in controlling thyroid cancer progression features, the specific EMT-related molecular mechanisms underlying OPN expression in thyroid tumors are still largely unknown.

This review aims at shedding light on the current knowledge related to EMT in thyroid tumors and the pivotal contribution of OPN as an EMT modulator in different tumor types. Considering these data, we focus on describing and discussing the putative relationship of OPN as a potential EMT regulator in the development of thyroid cancer, providing new clues regarding the molecular mechanisms by which OPN could modulate epithelial–mesenchymal plasticity in this tumor type, possibly generating evidence that can contribute towards the future development of new therapeutic, prognostic and/or diagnostic tools.

## 2. Thyroid Cancer

Approximately 90% of all thyroid cancers are differentiated thyroid carcinomas, which comprise papillary and follicular thyroid cancer. Papillary thyroid cancer displays mutually exclusive mutations of genes encoding effectors that signal through the mitogen-activated protein kinase (MAPK) and phosphoinositide 3-kinase/AKT serine/threonine kinase (PI3K/AKT) pathways. B-Raf proto-oncogene, serine/threonine kinase (*BRAF*) V600E accounts for about 60% of these mutations. In addition, mutations in RAS proto-oncogene, GTPase (*RAS*) and chromosomal rearrangements lead to sustained activation of the *BRAF* kinase domains or of tyrosine kinase receptors (TKRs), such as rearranged during transfection (*RET*), neurotrophic receptor tyrosine kinase (*NRTK)*, and anaplastic lymphoma receptor tyrosine kinase (*ALK*) [11].

Papillary thyroid cancer is the most common subtype and most of these patients have a good clinical outcome. However, the biology of this tumor type is extremely diverse, ranging from nonprogressive/extremely indolent lesions to aggressive metastatic tumors [12]. Data from patients carrying the *BRAF* V600E mutation, for instance, are controversial. Although the *BRAF* V600E mutation in papillary thyroid cancer is generally associated with aggressive phenotypes [13], higher rates of disease recurrence [14] and shorter disease-free and overall survival [13], this mutation cannot be considered as an independent poor-outcome prognostic factor [15]; however, it should be evaluated in association with other prognostic factors [16]. Follicular thyroid cancers represents 2 to 5% of thyroid cancer cases [17]. Follicular thyroid cancer and the follicular variant of papillary thyroid carcinoma are associated with mutually exclusive mutations of *RAS* or the paired box 8-peroxisome proliferator-activated receptor gamma (*PAX8–PPARG*) fusion oncogene [18]. Poorly differentiated and anaplastic types represent approximately 4% and 2% of all cases, respectively. Although rare, they represent the most aggressive thyroid tumors and are likely to be associated with lymph node and distant metastases [19]. During the process of tumoral transformation, they progressively lose thyroid typical cell features, becoming completely undifferentiated thyroid cancers, while poorly differentiated variants show an intermediate spectrum of differentiation [20,21].

It was shown that anaplastic, and to a lesser extent, poorly differentiated thyroid tumors, are characterized by the accumulation of different oncogenic alterations [22]. In both cases, the two most frequently altered genes are the telomerase reverse transcriptase (*TERT*) oncogene and the tumor protein 53 (*TP53*) tumor suppressor gene. Other gene mutations impair the MAPK and PI3K/AKT pathways, including in the *BRAF*, *RAS*, *AKT*, phosphatase and tensin homolog (*PTEN*), and phosphatidylinositol-4,5-bisphosphate 3-kinase catalytic subunit alpha (*PIK3CA*) genes, which were also reported to have a prevalence above 10% in at least one of the two histotypes [22].

The prevalence of the medullary type is approximately 3–5% of all thyroid tumors [2], accounting for 13.4% of the total deaths attributable to these malignancies [23]. Medullary thyroid cancer can be inherited or sporadic, in 25% and 75% of cases, respectively, with hereditary medullary thyroid cancer being part of the multiple endocrine neoplasia type 2 (MEN 2) syndrome [24]. Germline mutations of the *RET* proto-oncogene generate hereditary medullary thyroid cancer, whereas somatic *RET* and *RAS* mutations were described in this sporadic disease [2].

## 3. EMT, MET and Epithelial–Mesenchymal Plasticity 

During the last decades, once its aggressive subtypes were related to poor prognosis, short overall survival and therapeutic resistance, studies begun to explore the mechanisms underlying thyroid tumor progression [25]. A key event related to thyroid cancer progression is the EMT [10].

During cancer progression, tumor cells with epithelial features generally acquire mesenchymal traits through increased expression of EMT-related transcription factors (EMT-TFs), such as the twist family bHLH transcription factor (TWIST), snail family transcriptional repressor 1 (SNAIL), snail family transcriptional repressor 2 (SLUG) and zinc finger E-box binding homeobox 1 (ZEB), the expression levels of which vary according to tumor type. The expression of these transcription factors, along with the contribution of microRNAs (miRNAs), as well as epigenetic modifications, provide aggressive features to tumor cells by often repressing the expression of epithelial markers, such as E-cadherin and cytokeratin, while upregulating mesenchymal markers, such as N-cadherin and vimentin [3].

Through these modifications, cells tend to alter their epithelial-like phenotype by losing part of their apical-basal polarity and cell–cell adhesion, and acquire mesenchymal morphology features such as a spindle-like shape. Furthermore, cells reorganize their actin cytoskeletal features and upregulate the expression of matrix metalloproteinases, favoring migration and invasion into the surrounding tissues and lymph nodes, thereby becoming able to generate distant metastases. When tumor cells reach metastatic sites, they generally return to the outstanding epithelial phenotype in order to colonize new tissues through the EMT reverse process, MET, which is also a key event during the development of cancer [26].

Many studies show that the EMT/MET processes display great plasticity and reversibility, in which cells can switch to diverse and dynamic phenotypes, depending on the microenvironmental conditions and different tumor types [27]. Recently, it has become increasingly demonstrated that tumor cells usually simultaneously express both epithelial and mesenchymal markers, generating a substantial spectrum of a so-called intermediate or partial EMT phenotypes [3,4]. Indeed, recent reports have referred to the reversibility of the EMT and MET processes as epithelial–mesenchymal plasticity, in order to comprise the variations and the complex phenotype spectrums underlying these programs [28].

In addition to modulating tumor-progression features, such as migration and invasion, the EMT process has, so far, been related to the generation of cancer stem cells through the upregulation of stemness-related genes by EMT-related transcription factors [4]. Cancer stem cells are characterized as a tumor cell subpopulation that displays great self-renewal ability, high proliferation rates and high capacity to generate new tumors that recapitulate the heterogeneity of a primary tumor, once they can differentiate into diverse cell types [29]. EMT-related cancer stem cell generation consists of an important step of metastatic colonization, once EMT can modulate the expression of self-renewing and high-proliferation-associated genes, which it is thought to play a pivotal role in the establishment of distant metastases [4]. The expression of stemness-related genes can also generate the repression of EMT-related transcription factors, inducing the MET process, in addition to mediating therapeutic resistance, which is related, for instance, to the dynamic phenotypic plasticity, since the intermediate phenotypes of EMT are associated with efficient metastatic colonization and with higher rates of generation of cancer stem cells [30].

## 4. EMT in Thyroid Cancer

### 4.1. EMT-Related Transcription Factors Modulating the Expression of EMT Markers in Thyroid Cancer

In the papillary, follicular, anaplastic and medullary subtypes, the mechanisms of EMT have been extensively explored. Table 1 summarizes several examples of EMT modulating different thyroid cancer progression features, including migratory and invasive properties, the expression of EMT-related transcription factors and signaling pathways, the downregulation of epithelial markers, the upregulation of mesenchymal-associated genes, stemness, and therapeutic resistance, emphasizing how this process can modulate the aggressive properties of different thyroid tumors.

Specific mutations in thyroid cancer cells, such as *BRAF* V600E, have been described as EMT modulators, enhancing thyroid cancer progression features. For instance, increased cell invasion can be mediated by the *BRAF* mutation-induced activation of phosphorylated extracellular-signal-regulated kinase (pERK), phosphorylated mitogen-activated protein kinase (pMEK), nuclear factor kappa-light-chain-enhancer of activated B cells (NF-κB), and metalloproteinases activity [31]. The proto-oncogene *RET*, also highly expressed in papillary cancer, when overactivated, induces invasion and evades apoptosis by activating EGFR signaling pathway and mediating the process of EMT [44].

Further highlighting well-described pivotal role of EMT in the progression of thyroid cancer, Vasko et al. [32] reported that epithelial mesenchymal transition occurred in papillary thyroid carcinoma through the upregulation of vimentin levels in comparison to normal thyroid tissues, which was associated with metastatic and invasive samples. In anaplastic thyroid-derived cell lines, vimentin expression has also been associated with enhanced cell invasion. The expression of RUNX family transcription factor 2 (RUNX2) also represents a great example of EMT modulating the progression of thyroid cancer, since it is a thyroid transcription factor that is related to the induction of EMT [45], and it is also associated with the expression of vimentin and the occurrence of metastasis [32].

The EMT process tends to occur in the tumor-invasive area, where cells disseminate through the surrounding tissues. Papillary thyroid tumors presenting loss of cell cohesiveness in the invasive front display decreased expression of thyroid transcription factor-1 (TFF-1) and membrane E-cadherin levels, in addition to higher β-catenin and vimentin expression [34]. The presence of the invasive phenotype, a hallmark from EMT, in the thyroid cancer front has also been related to advanced tumors, lymph node metastases and tumor recurrence [34,46]. In addition to being downregulated in the tumor-invasive front, E-cadherin is also reported to be highly expressed in small papillary thyroid microcarcinomas [47].

Moreover, the expression of EMT-related transcription factors is thoroughly described in thyroid tumors, representing a major EMT molecular mechanism. In papillary and follicular tumors, TWIST, SNAIL and SLUG have been related to cell invasion and poor disease-free survival. E-cadherin and cytokeratin-19 had decreased expression while TWIST and vimentin were upregulated [37,43]. In the widely invasive follicular thyroid cancer, and in poorly differentiated and anaplastic carcinomas, these transcription factors were also upregulated. Specifically in invasive follicular thyroid carcinoma, high TWIST levels and downregulation of E-cadherin were significantly correlated with vascular invasion and poor disease-free survival [43]. TWIST1 and SLUG levels were upregulated in anaplastic in comparison to papillary and follicular thyroid tumor samples, while their expression was associated with decreased E-cadherin expression and metastatic tumors [48]. 

ZEB1 is also a transcription factor that is related to the activation of EMT with outstanding expression in thyroid tumors, with this being related to the occurrence of metastasis as well as cell migration, invasion and proliferation in anaplastic and in medullary thyroid carcinoma cell lines [49]. ZEB1 showed higher levels, as opposed to E-cadherin lower levels, in anaplastic thyroid cancer when compared to papillary and follicular thyroid cancer [35]. The SMAD transcription factor’s roles are further reported in thyroid tumors. High levels of SMAD family member 4 (SMAD4) were observed in follicular thyroid cancer [50], while SMAD family member 7 (SMAD7) was the most expressed transcription factor in anaplastic thyroid cancer [35,50]. The transcription factor Paired related homeobox 1 (PRRX1) also showed high expression levels in papillary derived cell lines and in tumors in which EMT was induced by transforming growth factor beta 1 (TGFβ1) treatment. In comparison to papillary samples, PRRX1 was upregulated in anaplastic thyroid cancer [37].

Unlike differentiated and anaplastic thyroid carcinomas, EMT pathways and mechanisms have not been much addressed in medullary and poorly differentiated thyroid cancers. However, it is known that EMT is important to medullary thyroid tumor progression, as the expression of C-X-C motif chemokine receptor 4 (CXCR4), which can activate EMT-pathways, was increased in advanced stages and larger tumors, as well as being associated with poor overall survival. Treatment with its ligand, C-X-C motif chemokine ligand 12 (SDF1α), induced cell invasion and higher bone marrow stromal cell antigen 2 (BST2), fibroblast growth factor 9 (FGF9) and vimentin levels, while E-cadherin levels were decreased [41].

Corroborating EMT-associated chemotherapy resistance, when treated with the *BRAF* inhibitor Vemurafenib, the 8505c cell line, which is derived from anaplastic thyroid carcinoma and is resistant to the this inhibitor, presented a mesenchymal-like morphology, higher migration rates and higher levels of phosphorylated MET proto-oncogene, receptor tyrosine kinase (p-c-MET), phosphorylated AKT (p-AKT), vimentin, β-catenin and CD44 protein when compared to inhibitor-sensitive papillary thyroid cancer cells [40]. Furthermore, along with EMT-induced tumor progression, thyroid tumor development has also been associated with cancer stem cell features. Anaplastic, follicular and papillary thyroid cancers presented high expression of stemness-related genes, such as POU class 5 homeobox 1 (*OCT4*), nanog homeobox (*NANOG*), ATP-binding cassette subfamily G member 2 (*ABCG2*) and stage-specific embryonic antigen 1 (*SSEA-1*), when compared to benign lesions and normal thyroid, which were enhanced by Fluorouracil treatment, indicating their possible additional role in tumor chemoresistance [38].

### 4.2. MiRNAs as Regulators of EMT in Thyroid Cancer

In EMT, miRNAs also have marked relevance in terms of the modulation of cell migration, invasion and metastasis. Several miRNAs have been found to be differentially regulated in thyroid tumor samples. Studies carried out on papillary thyroid cancer revealed the overexpression of miR-221, miR-222 and miR-146b [51] and the downregulation of miR-144 [52]. Among these, some have been described as inducers of EMT in thyroid tumor progression, such as miR-146b, which is responsible for high TWIST levels, the inhibition of E-cadherin and the downregulation of PTEN expression, leading to enhanced thyroid tumor cell proliferation, migration and invasion, in addition to the inhibition of apoptosis, through the upregulation of the PI3K/AKT pathway [53].

Moreover, in papillary thyroid cancer, high miR-221 expression was associated with EMT activation once it was related to the occurrence of lymph node metastasis, as well as enhanced cell invasion and migration along with high levels of vimentin, N-cadherin and SNAIL [54]. MiR-144-3p also proved to be an EMT inducer in papillary thyroid cancer, as it was responsible for decreased E-cadherin and thyroid-specific transcription factor PAX8 expression while upregulating vimentin and N-cadherin. It also induced AKT, ERK and mitogen-activated protein kinase 8 (JNK) signaling, in addition to causing radio and chemotherapy resistance [55]. 

In parallel, there are also tumor suppressor miRNAs that inhibit thyroid cancer progression and the EMT process. For instance, miR-31 presents low expression levels in papillary thyroid cancer, while its overexpression promotes vimentin, N-cadherin, p-AKT and p-ERK downregulation, as well as decreased cell migration and invasion [56]. Similarly, the overexpression of miR-520a-3p also upregulated E-cadherin, and decreased the expression of N-cadherin and vimentin, while inhibiting cell migration and invasion in papillary thyroid cancer [57]. 

Anaplastic thyroid cancer also displays deregulation in miRNA levels. According to the evidence that relates anaplastic development to differentiated thyroid cancer dedifferentiation and EMT upregulation [58], the miR-200 and miR-30 families, in addition to E-cadherin expression, were decreased, while ZEB1/2, SMAD2 and transforming growth factor beta receptor 1 (TGFBR1) were upregulated in anaplastic thyroid cancer, when compared to papillary and follicular thyroid cancer. In addition, miR-200 and miR-30 overexpression in anaplastic thyroid cancer induced high E-cadherin levels as well as the downregulation of vimentin, N-cadherin, ZEB1/2, SMAD2 and TFGBR1, in addition to the inhibition of cell invasion, highlighting their role as possible MET and differentiation regulators [33].

## 5. Osteopontin Expression and Crosstalks with EMT in Thyroid Cancer

Osteopontin is encoded by the secreted phosphoprotein 1 (*SPP1*) gene [7] and is expressed by a variety of cells, including epithelial cells and cells from the tumor microenvironment, tumor stroma and the inflammatory niche (such as neutrophils, natural killers, leucocytes, macrophages and T cells). OPN secreted from the stromal cells is responsible for the recruitment and activation of inflammatory cells, increasing cell invasion and migration and evasion of the immune system [59]. This protein was described as a prime regulator of EMT process [5]. OPN is mostly described as having a secreted form, although intracellular variants have also been reported [60]. In its protein structure, there are specific domains of interaction with integrins and CD44 receptors, modulating the activation of diverse cell functions, such as survival, angiogenesis, proliferation, and migration [61]. In addition to several pathobiological roles, osteopontin can act as an immune response modulator and is often described as a cancer-related protein [62]. Through interaction with its receptors, osteopontin and its most-described splicing variants can activate signaling cascades, such as PI3K/AKT and MAPK/ERK, leading to the modulation of EMT-related transcription factor and gene expression, which has been described in several tumor types such as melanoma, breast, ovarian, liver, hepatocellular, colorectal and gastric cancers [5,61]. 

Regarding the OPN splicing variants’ roles in the EMT process, high OPNa and OPNc expression were related to high MMP-9 levels in metastatic samples of hepatocellular carcinoma [63]. In addition, OPNb and OPNc were described as important modulators of chemotherapy resistance and EMT induction. In prostate cancer, cells overexpressing OPNb and OPNc, upon docetaxel treatment, had lower E-cadherin, claudin and cytokeratin levels, while N-cadherin, vimentin, SNAIL, SLUG and TWIST were upregulated in comparison to cells overexpressing OPNa and empty vector transfected cells [47]. In non-small cell lung carcinoma, OPN was also reported to modulate the EMT process. The overexpression of OPNc was related to higher migration and invasion rates, downregulation of E-cadherin and enhanced expression of N-cadherin, in comparison to the overexpression of OPNa and OPNb [64]. 

Remarkably, a recent study showed that OPN can modulate not only EMT activations, but also the so called epithelial mesenchymal plasticity, through the opposite functions of secreted OPN and intracellular OPN [65]. In some hepatocellular carcinoma cell lines, intracellular only, but not secreted OPN, was expressed while its overexpression was capable of inducing MET features, through the upregulation of E-cadherin and the decrease of mesenchymal markers such as vimentin, N-cadherin, alpha-smooth muscle actin (α-SMA), ZEB1 and ZEB2. This work also demonstrated that intracellular OPN-induced mesenchymal epithelial transition was dependent on miR-429 and AKT1 expression and endothelial PAS domain protein 1 (HIF2α) downregulation, in addition to being pivotal to the establishment of lung metastasis, while in other cancer cell lines that expressed secreted OPN, the secreted OPN induced EMT-related tumor-progression features [65]. 

In thyroid cancer, OPN messenger RNA (mRNA) expression was first described in papillary thyroid cancer samples, specifically in tumor-associated macrophages [66], presenting higher levels when compared to the corresponding normal thyroid [66,67,68]. OPN expression has been found in distinct thyroid cancer types, including papillary, follicular, anaplastic and medullary thyroid cancer, and is overexpressed in thyroid tumor tissues in comparison to benign and normal adjacent tumor samples, with the exception of medullary thyroid cancer. Table 2 summarizes these data.

Papillary thyroid tumors are subdivided into well-characterized variants [84]. Among these, OPN expression was evaluated in the classic variant of papillary thyroid cancer, as well as in the follicular variant and the tall cell variant [9,71,74,76]. The classic subtype, known for its papillae morphology and the presence of psammoma bodies, displays high OPN levels [71,76], which are related to vascular invasion [9]. The follicular variant, characterized by most cells presenting follicular shape with no well-formed papillae and similar prognosis as the classic variant, is related to lower OPN expression and to the absence of lymph node metastases [76,84]. The tall cell variant, which displays most cells being more tall than wide and usually comprises an aggressive phenotype, also exhibited high OPN levels in relation to the follicular variant and to normal thyroid tissues [71,76].

Furthermore, papillary thyroid cancer samples harboring the -443C>T polymorphism in the OPN promoter region are associated with higher papillary thyroid cancer risk and presented higher OPN levels than in tissues lacking this genotype [77]. Park et al. [85] also described OPN high levels in papillary thyroid carcinomas, which were significantly associated with advanced tumor stages. When the papillary type was combined with the occurrence of Hashimoto’s thyroiditis, OPN plasma levels and tissue expression were decreased compared to the results found in papillary cancer alone. In terms of the regulation of OPN expression, in papillary thyroid carcinomas, an association was found between the OPN-encoding gene *SPP1* and the high-mobility group proteins AT-hook 1b (HMGA1b) and chromobox homolog 7 (CBX7), which upregulated and downregulated *SPP1* expression, respectively, through specifically binding to its promoter [78]. 

The first description of OPN in follicular thyroid cancer reports OPN similar levels for both follicular and papillary thyroid carcinomas, with slightly higher expression in papillary thyroid cancer [72]. Indeed, most authors observed the papillary type presenting higher levels when compared with follicular thyroid cancer [9,73,76]. In transgenic mice harboring aggressive follicular thyroid cancer, tumors also presented high OPN levels [83]. For medullary thyroid cancer and, especially, anaplastic thyroid cancer, OPN protein levels were enhanced in comparison to papillary thyroid cancer tissues, which were increased in medullary and anaplastic metastatic samples [73].

Among the reports describing OPN expression in thyroid tumors, our group was the first to describe splice variants of osteopontin expression in thyroid tissues. We found that OPNa was the most expressed splice variant in the classic and follicular variants of papillary cancer and in follicular thyroid cancer, as well as in benign and normal thyroid tissues, although classic PTC presented the highest OPNa levels. In thyroid cancer cell lines, OPNa was also the most expressed OPN-SV, followed by OPNb [9]. Consistently, further studies showed that the OPNb variant exhibited high transcript levels in metastatic papillary thyroid carcinoma and was associated with integrin α5 expression, whereas information about the osteopontin expression levels in other splice variants is lacking [81].

Notably, medullary thyroid carcinoma presented an opposite OPN expression pattern in comparison to follicular-derived thyroid cancers; it was found that OPN was associated with small and non-invasive tumors, in addition to decreased cell proliferation and viability, in medullary thyroid carcinoma cell lines. OPNa was also the OPN’s splice variant with the highest expression in patient samples and medullary thyroid carcinoma-derived cell lines. However, total OPN and OPNa had high levels not only in medullary thyroid carcinoma, but even more in the normal thyroid C-cells and in C-cell hyperplasia, a benign C-cell cluster, respectively, while in normal thyroid follicular cells, OPN expression was absent [80]. 

In addition to the known participation of OPN in EMT, when taking together data regarding OPN in thyroid cancer progression (Table 2) and evidence on the main contribution of EMT in thyroid tumors [10] (Table 1), the literature set enables the discussion of related molecular mechanisms whereby OPN potentially induces EMT in this tumor model. 

The overexpression of OPN in thyroid tumor cells has been already related to diverse tumor progression steps such as migration and invasion [9], which are known EMT-mediated tumor hallmarks [3]. In papillary thyroid carcinomas, the expression of OPN is also enhanced with increasing malignancy [73], and has been correlated with lymph node metastasis, tumor size, advanced stage tumors and vascular invasion [9,76,77]. In medullary and anaplastic thyroid cancer, lymph node metastases also showed higher OPN expression in relation to their primary tumor [73]. In classic PTC, OPN levels are also associated with tumor calcification [86] which can be related to the occurrence of EMT features, once papillary thyroid cancers harboring calcification are frequently associated with lymph node metastasis [82]. 

OPN plays key roles in the modulation of specific signaling pathways activated by genetic changes in thyroid cells [69]. OPN treatment in cells overexpressing *RET/PTC* enhanced cell proliferation, invasion and dissemination while high OPN expression was related to RAS/MAPK signaling activation, since the overexpression of the v-Ha-Ras variant upregulated OPN levels while MEK1 inhibition promoted the downregulation of OPN. *RET/PTC* rearrangements were also associated with high CD44 expression while OPN-mediated cell dissemination was decreased by the inhibition of CD44 [69], suggesting that OPN may have a complementary role in the progression of papillary thyroid cancer through the interaction with its known receptor CD44, which is related to tumor-progression features [61]. Indeed, De Falco et al. [87] found that CD44 cleavage and the activation of thyroid cancer cells’ proliferation by its fragment CD44-ICD appears to be mediated by the expression of *RET/PTC*, which might illustrate a putative link in terms of their associated expression. 

Other reports also associate the overexpression of osteopontin to CD44 high levels and the activation of MAPK signaling. Guarino et al. [71] showed that classic papillary thyroid cancer samples and papillary thyroid cancer cell lines had high OPN and CD44v6 variant expression. Osteopontin treatment increased CD44 and p-44/42 MAPK levels, as well as inducing higher invasion rates, which were inhibited by CD44 and MAPK pathway repressions. Moreover, diverse papillary thyroid cancer cell lines harboring *RET/PTC* rearrangements or *BRAF* V600E mutation had higher osteopontin expression in comparison to normal thyroid cells. Papillary thyroid cancer samples harboring the *BRAF* V600E mutation, or *RET/PTC1* or *RET/PTC3* rearrangements, also had higher vimentin, RUNX2 and osteopontin expression in relation to non-mutated samples [32], once again highlighting the importance of these genetic alterations to the putative OPN-mediated EMT in papillary thyroid cancer progression. Osteopontin expression in papillary thyroid cancer was also correlated with phosphorylated-JNK (p-JNK) expression [74], which is a MAPK subfamily kinase related to aggressive papillary thyroid cancer [88]. Thus, osteopontin expression may be associated with the activation of EMT through the upregulation of the *RET/PTC*/RAS/MAPK signaling cascade. 

Moreover, OPN can be related to EMT-associated RAS/MAPK activation through the expression of *BRAF* [76,89]; in papillary thyroid cancer, high OPN expression was associated with the *BRAF* V599E mutation along with advanced tumors and lymph node metastasis [76], and also with *BRAF* V600E mutated tumors [32,71]. Associations of osteopontin expression and mutated *BRAF* may represent another prospective source of evidence according to which osteopontin could be inducing EMT in thyroid cancer, whereby *BRAF* mutation, especially *BRAF* V600E, is closely related to EMT activation [90,91]. The activation of EMT-related genes by *BRAF* V600E was also remarkably associated with well-differentiated papillary progression to poorly differentiated and anaplastic thyroid cancer [90]

The activation of PI3K/AKT signaling in papillary thyroid cancer was also related to the overexpression of osteopontin, which induced upregulation of p-AKT levels. Both MAPK and AKT activation were decreased by the inhibition of CD44. Further, OPN-induced cell invasion was AKT-dependent [71]. *RET/PTC1* transformed thyroid cells displaying high proliferation rates and high p-AKT, p-ERK1/2, MMP-2 and MMP-9 expression also exhibited higher *SPP1* expression in relation to normal and *RET/PTC1* mutant thyrocytes [70]. In follicular thyroid cancer aggressive tumors, along with high expression of osteopontin, increased levels of p-AKT were also observed, which may be associated with the integrin-activated PI3K/AKT signaling pathway as, in addition to high expression of osteopontin and p-AKT, integrin β1 also showed increased levels [83]. These data highlight the activation of the PI3K/AKT cascade as one of the putative pathways by which OPN could modulate the EMT process in thyroid tumor progression.

In addition to high levels of osteopontin, p-AKT and integrin β1, TGFβ and tumor necrosis factor-alpha (TNFα) exhibited an outstanding expression in follicular thyroid cancer [83]. TGFβ proteins can activate EMT signaling cascades during the progression of thyroid cancer [92], while TNFα can also activate EMT [4]. TGFβ and TNFα can also regulate the expression of osteopontin [93]. TNFα-induced osteopontin expression is integrin-β1-dependent, as well as being MAPK-dependent [94]. These data suggest an additional possible mechanism relating OPN-mediated EMT to thyroid cancer.

NF-κB signaling cascade activation is another pathway reported to be associated with OPN expression in thyroid tumors, once follicular thyroid cancer displaying high OPN expression also exhibited high levels of the p65 NF-κB subunit [83], while, in papillary thyroid cancer, the overexpression of NF-κB increased *SPP1* expression, which was modulated by the HGMA1b and CBX7 proteins [78]. HMGA1b, which upregulated *SPP1* expression and NF-κB activity [78], is further reported to be activated by TGFβ1 in a PI3K/AKT and MAPK-dependent manner and to play an important role in positively modulating thyroid tumor cell invasion and migration, in addition to upregulating the expression of metalloproteinases and decreasing E-cadherin levels [95].

Regarding the expression of OPN’s splice variants in relation to EMT in thyroid cancer, osteopontin, specifically the OPNa variant, is reported to be upregulated in papillary thyroid cancer samples when compared to non-tumoral tissues and to be related to larger tumor size and invasive features. In addition, the overexpression of OPNa resulted in higher cell proliferation, migration, invasion and metalloproteinases activation in follicular-derived cell lines [9]. More recently, we found that thyroid cancer cells overexpressing OPNa can increase vimentin expression and induce cytoskeleton remodeling, as well as favoring several stemness features (Gimba et al., 2020; data not published yet). 

Otherwise, we showed that, unlike the well-described role of OPN in the modulation of tumor-progression features [5], in medullary thyroid cancer, overexpression of the OPNa variant is associated with cell differentiation and with good prognosis, and its expression was enhanced in non-invasive and small sized tumors, while its ectopic overexpression in medullary thyroid cancer cell lines induced lower proliferation and viability rates [80]. Moreover, the overexpression of OPNa induced high expression of specific thyroid C-cell-related genes and epithelial-like features in medullary thyroid cancer cell lines, such as increased cell–cell adhesion as well as the downregulation of TWIST expression and decreased cell migration (Viana et al., data not published). These data suggest that OPN may have a key role in modulating the MET process in medullary thyroid cancer, instead of inducing a mesenchymal-like phenotype, of which we continue to explore the underlying molecular mechanisms that are yet to be elucidated.

## 6. Final Statements and Concluding Remarks

**Figure 1:**
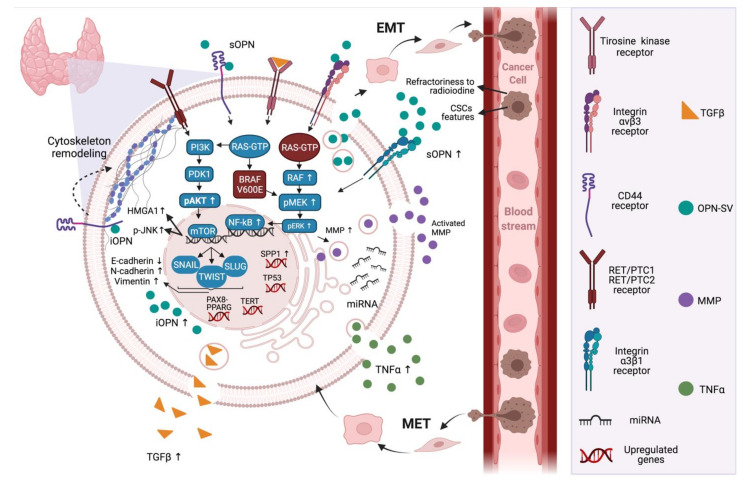
OPN signaling in the context of epithelial–mesenchymal plasticity in thyroid cancer cells. In thyroid cancer cells, OPN is a highly expressed protein that plays major roles in the regulation of the PI3K/AKT and MAPK signaling pathways. OPN can induce these pathways through interacting with its known receptors, CD44 and/or integrins. In thyroid cancer, the expression of OPN is related to mutated *RAS* and *BRAF* genes, in addition to chromosomal rearrangements of *RET*. These pathways lead to the expression of transcription factors, including NF-κβ, SLUG, SNAIL and TWIST, which are correlated with cellular plasticity and tumor progression. Further, other genes are likely to be mutated in thyroid cancer, such as the oncogene *TERT*, *PAX8-PPARG* fusion and the tumor suppressor gene *TP53*. Furthermore, the inducing of transcription factors related to EMT, OPN-SV plays direct roles in activating extracellular MMPs by binding to integrin receptors and activating signaling pathways including MAPK. Additionally, intracellular OPN can regulate cytoskeleton remodeling through the CD44 receptor, which are pivotal steps to increased cell migration, invasion and metastases. In addition, TNFα and TGFβ can be regulated by OPN and can, in turn, regulate the expression of OPN. OPN seems to also be a target for miRNAs, which are related to the regulation of the EMT process. Moreover, the expression of OPN is correlated with the expression of p-JNK and HMGA1b. In thyroid cancer, OPNa is the most expressed OPN-SV and potentially induces EMT. As a result of these events, thyroid cancer follicular cells acquire mesenchymal features, through the downregulation of E-cadherin and the upregulation of mesenchymal markers such as N-cadherin and vimentin, which are pivotal to the establishment of metastasis and refractoriness to radioiodine. Mutations in thyroid cancer are expressed differently among the subtypes of cancer, which causes the diversity of prognostics and overall survival. Up arrows mean levels increase and down arrows mean levels decrease. Created with BioRender.com (accessed on 28 July 2021).

Given the overexpression of osteopontin in thyroid cancer cells and its known association with several events that are generally associated with EMT in thyroid tumors, these data further reinforce the notion that OPN is also a master regulator of epithelial mesenchymal plasticity in thyroid cancer. Figure 1 summarizes OPN expression patterns and the signaling pathways that are altered during the EMT and MET processes, and their respective genetic modifications. Further characterization of how OPN modulate these molecular hallmarks can open new avenues to control the EMT processes that are classically involved in tumor invasiveness and metastatic potential in thyroid cancer.

## Figures and Tables

**Table 1:** EMT modulates diverse thyroid cancer progression stages.

Thyroid Tumor Model	EMT-Associated Biological Role	Bibliographic Reference
FTC cell line (WRO) PTC cell line (NPA) ATC cell line (KTC-3)	Induced *BRAF* V600E mutation expression was related to high pMEK, pERK, NF-κB and MMPs expression and increased cell invasion	[31]
PTC samples PTC cell line (NPA) ATC cell line (ARO)	In papillary thyroid carcinoma, RUNX2 and fibronectin1 had high expression in the tumor invasive area. Also, vimentin presented high levels in PTC, which was related to cell invasion and metastatic tumors	[32]
ATC, PTC and FTC samples ATC-derived cells	In ATC samples, miR-200 and miR-30 were decreased while ZEB1/2 and SMAD2 were upregulated in ATC samples in comparison to papillary and follicular tumors. Also, miR-200 and miR-30 overexpression in ATC induced an epithelial-like phenotype, harboring low vimentin levels and decreased cell invasion	[33]
PTC samples	Cells in papillary tumors invasive front with loss of cohesiveness and cell polarity had decreased membrane E-cadherin expression and were related to lymph node metastases	[34]
cPTC, FV-PTC, FTC and ATC samples PTC cell line (TPC-1)	Anaplastic tumors were related to decreased survival rates, high ZEB1 and SMAD7 and low E-cadherin expression in comparison to papillary and follicular carcinomas	[35]
ATC cell lines (ACT-1, FRO, KTC-2 and KTC-3) PTC cell line (TPC-1)	SNAIL overexpression in cell lines derived from anaplastic carcinomas generated E-cadherin downregulation, vimentin high expression and enhanced cell migration	[36]
PTC cell lines (BCPAP and TPC-1) ATC cell lines (THJ-16T and THJ-21T)	TGFβ treatment induced PRRX1, TWIST, SLUG, SNAIL and vimentin high expression and E-cadherin and cytokeratin 19 downregulation in papillary carcinoma derived cell lines, besides stemness properties and efficient tumor formation in mice	[37]
PTC samples PTC cell line (TPC-1) FTC cell lines (ML1, FTC236 and FTC 238) ATC cell lines (T238 and SW1736)	Stemness-related genes *OCT4*, *ABCG2*, *CD44*, *NANOG*, and *SSEA-1* exhibited high expression in thyroid tumor cells when compared to normal thyroid, which were upregulated by Fluorouracil chemotherapy treatment	[38]
PTC tissue samples PTC cell lines (IHH-4, TPC1 and BCPAP)	In papillary thyroid carcinoma, TWIST1 exhibited high expression in comparison to normal adjacent thyroid cells and was correlated to lymph node metastasis, cell invasion and vimentin expression	[39]
PTC cell line (BCPAP) ATC cell line (8505c) Athymic nude BALB/c mice	ATC cells showed enhanced cell migration and high p-c-MET, p-AKT, vimentin, β-catenin and CD44 expression, besides high tumorigenicity, upon *BRAF* V600E inhibiton, demonstrating EMT-related chemoresistance	[40]
MTC samples MTC cell line (TT)	The chemokine receptor CXCR4 expression was high in medullary thyroid carcinoma in comparison to normal thyroid cells, which was also related to bigger and advanced stage tumors and lymph node metastasis. Cell treatment with chemokine SDF1α, ligand to CXCR4, enhanced MTC cell invasion	[41]
Papillary thyroid microcarcinoma (PTMC) samples	Stemness genes *ALDH1A3*, *TM4SF1*, *PROM1* and EMT-associated *CAV1* gene had high expression in PMTC with neck-node metastases in comparison with non-metastatic papillary microcarcinomas	[42]
FTC, PDTC and ATC samples	SNAIL, SLUG and TWIST had high expression in widely invasive FTC, ATC and PDTC. Also, high TWIST levels were correlated with vascular invasion and poor disease-free survival	[43]

Abbreviations: ATC, anaplastic thyroid cancer; cPTC, classic variant of PTC; FTC, follicular thyroid cancer; FV-PTC, follicular variant of PTC; MTC, medullary thyroid cancer; PDTC, poorly differentiated thyroid cancer; PTC, papillary thyroid cancer; PTMC, papillary thyroid microcarcinoma.

**Table 2:** OPN expression is associated with thyroid cancer progression in distinct subtypes.

Thyroid Tumor Model	OPN Expression	EMT-Associated Biological Role	Bibliographic Reference
PCCL3, TPC-1 and FB2 cell lines	High OPN expression was related to *RET/PTC1*, *RET/PTC3*, CD44, HA-RAS and MEK1	OPN treatment generated increased cell proliferation, invasion and dissemination	[69]
Non-tumor thyroid cells overexpressing *RET/PTC1* PTC and FTC samples	High OPN expression in *RET/PTC1*-thyrocytes and in PTC samples	High OPN expression generated increased proliferation and high p-AKT, p-ERK1/2, MMP-2 and MMP-9	[70]
cPTC, FV-PTC and TCV-PTC samples PTC cell lines (TPC1, FB2, BHP2-7, BHP7-13, BHP10-3, BHP5-16, BHP14-9, BHP17-10, NPA and BCPAP)	OPN had higher expression in cPTC and TCV-PTC	OPN was related to lymph node metastasis, cell invasion and high levels of CD44, p44/42 MAPK and p-AKT	[71]
PTC and FTC samples FTC cell line (WRO) PTC cell line (NPA)	Higher OPN expression in PTC	OPN expression was correlated with the occurrence of lymph node metastasis	[72]
PTC, FTC, ATC and MTC samples	Tumor tissues had high OPN expression	MTC and ATC lymph node metastasis had higher OPN expression compared to their primary tumors	[73]
cPTC, FV-PTC and other PTC variants samples	OPN had higher expression in PTC	OPN expression was positively correlated with p-JNK	[74]
PTC samples	PTC samples had high OPN expression	OPN expression was related to microcalcification and lymph node metastasis	[75]
cPTC, FV-PTC and TCV-PTC samples	OPN had high expression in PTC	High OPN expression was related to lymph node metastasis, advanced N stage and *BRAF* V599E mutation	[76]
PTC samples	OPN -443 C>T polymorphism exhibited high expression in PTC	OPN -443 C>T was related to higher PTC and cervical lymph node metastasis risk and angiolymphatic invasion	[77]
PTC cell lines (B-CPAP and TPC1)	OPN expression was upregulated and downregulated by HGMA1b and CBX7, respectively	OPN expression induced increased cell migration	[78]
PTC samples	PTC samples exhibited high OPN expression	High OPN expression was related to calcification areas	[79]
MTC samples MTC cell lines (TT and MZ-CRC-1)	C-cells and C-cell hyperplasia exhibited higher OPN expression than MTC samplesOPNa was the most expressed OPN-SV	High expression of OPN in small, non-invasive tumors with PTEN nuclear expression and wild-type RAS	[80]
cPTC, FV-PTC and FTC samples PTC cell lines (TPC1, K1 and BCPAP) ATC cell lines (KAT4, Hth74, 8505c and c643)	cPTC exhibited higher OPN expression OPNa was the most expressed OPN-SV	OPNa expression was related to vascular invasion, extrathyroid extension, cell migration and invasion and MMPs activity	[9]
PTC samples	PTC lymph node metastasis samples showed OPNb high expression	OPNb and integrin α5had high expression in metastatic samples	[81]
cPTC samples PTC cell line (TPC1) ATC cell line (c643)	OPN exhibited high expression in cPTC samples Among OPN-SV, OPNa and OPNb were the most expressed	OPN expression in psammoma bodies was related to lymph node metastasis OPNa overexpression induced matrix calcification and collagen synthesis	[82]
Thrb^PV/PV^Pten^+/−^ mice with aggressive FTC	OPN showed high levels in Thrb^PV/PV^Pten^+/−^ mice	In Thrb^PV/PV^Pten^+/−^ tumors, OPN, integrin β1, pAKT, p65, TGF-β1 and TNF-α had high expression	[83]

Abbreviations: ATC, anaplastic thyroid cancer; cPTC, classic variant of PTC; FTC, follicular thyroid cancer; FV-PTC, follicular variant of PTC; MMPs, metalloproteinases; MTC, medullary thyroid cancer; PDTC, poorly differentiated thyroid cancer; PTC, papillary thyroid cancer; TCV-PTC, tall cell variant of PTC.
